# Thailand medical mobile application for patients triage base on criteria based dispatch protocol

**DOI:** 10.1186/s12911-020-1075-6

**Published:** 2020-04-09

**Authors:** Krongkarn Sutham, Pattaraporn Khuwuthyakorn, Orawit Thinnukool

**Affiliations:** 1 Department of Emergency Medicine, Faculty of Medicine, Chiang Mai University, Chiang Mai, 50200 Thailand; 2 Research Group of Embedded Systems and Mobile Application in Health Science, College of Arts, Media and Technology, Chiang Mai University, Chiang Mai, 50200 Thailand

**Keywords:** Triage, Emergency medical service, Primary medical, Incident dispatch code, Criteria-based dispatch, Mobile application development

## Abstract

**Background:**

Before patients are admitted into the emergency department, it is important to undertake a pre-hospital process, both in terms of treatment performance and a request for resources from an emergency unit. The existing system to triage patients in Thailand is not functioning to its full capacity in either the primary medical system or pre-hospital treatment with shortcomings in the areas of speed, features, and appropriate systems. There is a high possibility of issuing a false Initial Dispatch Code (IDC), which will cause the over or underutilisation of emergency resources, such as rescue teams, community hospitals and emergency medical volunteers.

**Methods:**

A usability system design, together with a reliability test, was applied to develop an application to optimise the pre-hospital process, specifically to sort patients, using an IDC to improve the request for emergency resources. The triage mobile application was developed on both iOS and Android operating systems to support patient triage based on Criteria Based Dispatch (CBD). The 25 main symptom categories covered by CBD were used to design and develop the application, and 12 emergency medical staff, including doctors and nurses, were asked to test the system in the aspects of triage protocol correction, triage reliability, usability and user satisfaction.

**Results:**

The results of testing the proposed triage application were compared with the time used to triage by experienced staff and it was found that, in non-trauma cases, it was faster and more effective to use the application for emergency operations and to correct the IDC code representation.

**Conclusions:**

The triage application will be utilised to support the pre-hospital process and to classify patients’ conditions before they are admitted to the Emergency Department (ED). The application is suitable for users who are not medical emergency staff. Patients with non-trauma symptoms may be a suitable group to use the application in terms of time used to identify IDC for their own symptoms. The use of the application can be beneficial for those who wish to self-identify their symptoms before requesting medical services.

## Background

The application of smart technology has become an increasingly common practice in daily life, especially in medical treatment**.** Modern smart technology facilitates faster computation, has natural**-**command abilities, wider presentation screens and intelligent operation [1–4]. Advanced smart technology has also been found to be widely used in medical healthcare; for example, in monitoring patients, tracing their health conditions, consultations by experts or doctors, obtaining suggestions related to healthcare medication, and much more [5, 6]. The number of people who need to consult doctors or receive medical services could be reduced by substituting new technology as an alternative [7, 8].

Although the trend of using smart technology in healthcare has increased on a global scale, thereby reducing the duties of medical staff, inadequate public health support and services are still problematic in Thailand. While applications of smart technology have been developed in an effort to ease the overcrowding of emergency care units, this has not solved all their operational problems.

According to the National Bureau of Statistics, there were 220 million out-patients in Thailand in 2018, who were served by 35,388 doctors, 180,589 service providers and various other hospital staff [9]. Based on these numbers, the Thai healthcare system should be better at aiding primary care doctors. An effective primary care regime should include a self-care system, a long-distance patient care system, an effective patient-screening system, and a channel to deliver self-care knowledge to alleviate the workload of the public health units. Technology could be used to effectively manage medical personnel, especially primary care doctors, and patients could be treated in the order of the available resources and medication. These are the key elements of the national strategic plan for public health, which will be implemented over 20 years based on policy 4.0 [10] of the Thai government.

However, the disproportion between doctors and patients is causing concern. There are many reasons for overcrowding in hospitals across Thailand; for example, it can be difficult to predict the symptoms of a disease, side effects are sometimes unclear and patients involved in an accident often require a quick assessment. Primary care physicians play an important role in assessing patients in order to provide them with appropriate and timely medical services, both for normal illnesses and emergencies [11, 12].

Pre-hospital information provided by rescue teams, rescuers, community hospitals and emergency medical volunteers is invalid in many cases due to the incorrect Initial Dispatch Code (IDC), which leads to over-allocating or under-allocating emergency resources. The National Institute of Emergency Medicine of Thailand has reported that 60% of the patients who come to the Emergency Department (ED) do not require urgent treatment. This issue especially occurs in regions in which patients need immediate care from medical providers because they lack confidence in their access to primary care [13, 14]. As a result, many patients are admitted to an emergency room, which leads to the overcrowding of emergency care units, including the Out Patient Department (OPD) and the ED. Therefore, an effective patient assessment during the pre-hospital process could reduce the overcrowding in hospitals. For example, in an emergency, the rescue teams, rescuers, community hospitals and emergency medical volunteers are responsible for assessing and analysing patients and requesting all the necessary emergency resources. When these medical professionals and volunteers have no experience of making a comprehensive health assessment, the classification of patients using Criteria Based Dispatch (CBD) could be misrepresented, leading to an incorrect IDC and the over- or under-utilisation of emergency resources.

The best way to correctly assess a potential patient is to ask the provincial emergency command unit CBD questions, which results in an IDC. This process can take at least 2–3 min or more by phone. When an IDC is identified, the provincial emergency command unit requests an emergency dispatch from the nearest hospital to collect the patient. Trying to reduce the time and optimise the performance of the pre-hospital process is a continuous challenge for the National Institute for Emergency Medicine of Thailand (NIEM). The systematic patient assessment programme it developed, which is currently used for patient assessment to triage patients for IDC, has been found to be too difficult to use because a computer is required to run the programme (Microsoft Access) and this is impractical for primary emergency medical unit operations. Hence, it is only used by the provincial emergency command units, which are the central emergency units. Due to its inefficiencies, this system can only be leveraged in central call centres and is not used in the field.

Moreover, most Thai medical doctors use imported medical applications. Popular systems like the Canadian Triage mobile application and the Mobile Emergency Severity Index (ESI), are the standard for the emergency medical service in Thailand. These applications are widely used by medical doctors for patient triage because they simplify triage protocol referencing by providing a mobile interface. Based on the results of the study, mobile applications may have improved benefits for medical professionals and volunteers experienced in triage [15]. However, these applications are restricted to users with medical knowledge due to their advanced medical terminology. This is important, since the pre-hospital process not only involves medical staff, but also volunteers with limited medical knowledge.

The purpose of this paper is to determine the kind of system that can provide optimal patient triaging for requesting an accurate IDC. Additionally, it is important to find a practical solution for real-life operations by rescue teams, rescuers, community hospitals and emergency medical volunteers.

Moreover, there are other concerns that should be considered when developing an optimised medial system, such as how the system can reduce the overcrowding of the ED, how it can increase patients’ knowledge when they discover suspicious symptoms, and whether they can assess those symptoms to determine if they need urgent treatment when they are at home.

Therefore, an ideal system is one that both primary care physicians and patients can use to screen illnesses in both normal and emergency cases. It should also be able to refer patients to appropriate emergency medical services by helping them to assess the severity of their symptoms and the need for urgent treatment. The system should enable patients to classify symptoms according to international standards.

The aim of this research it to develop a mobile medical application which is suitable for use in Thailand based on the aforementioned requirements. This application must be capable of enabling patients to triage medical conditions based on dispatch protocol-grade criteria. It must be suitable for use on mobile devices because it will serve as a tool for both primary emergency medical practitioners and general users. A prototype will be developed in this study by using patients’ assessment data from the NIEM. This application is expected to reduce the limitations of existing systems that require specialised knowledge to effectively triage patients.

### Triage medical System

The use of technology via smart devices has transformed many aspects of the clinical practices of healthcare professionals. Mobile devices such as tablets are now commonly used in healthcare, which has led to the rapid development of medical software applications (apps). Mobile technology plays a significant role in terms of patient care by enabling users to track their health condition and suggesting suitable medication, as well as providing tools for medical providers to monitor their patients [16, 17].

The use of systems based on smartphones facilitates the effective tracking and managing of patients’ health [1, 2, 18, 19]. A variety of research that has been conducted in relation to applications associated with emergency cases will be discussed in this section.

The incorporation of information technology into the worldwide medical field via smartphones has become increasingly common due to its accuracy. For example, based on Scott’s study, the application of electronic triage increased the accuracy of the triage protocol. The probability of clinical care, emergency surgery and hospitalisation was indicated when applying the e-triage predictor and algorithms [20].

Tadahiro recently developed a system to optimise emergency department operations. Since the prediction of the ED disposition at the triage level remains challenging, this system is expected to enhance it, as well as improve the ability to predict the disposition of patients and support various medical duties [21].

Lei and his team studied the use of triage to identify patients who require immediate resuscitation, assign them to a pre-designed patient care area, and administer the appropriate diagnostic/therapeutic measures based on the use of ESI in a paediatric emergency room. According to the results, nurses improved their performance, taking approximately 2 min for triage, which was similar to that of doctors for ESI. Patients in levels 1–3, who require immediate, life-saving intervention without delay are at great risk of deterioration due to time-critical problems. This causes an urgent demand for resources to investigate and treat them and, according to the findings of this study, nurses are able to identify severe paediatric cases [22, 23].

Moreover, triage mobile applications have been developed and applied to dental science. For instance, Corey developed a mobile application for triaging dental emergencies based on the need to analyse and assess captured intraoral images [24]. Patients are able to self-identify their dental problem and complete a triage report within 4 min by selecting the most appropriate scenario that describes their discomfort. This application helps both dentists and patients to save time prior to a visit.

In Thailand, Sumrumeram developed a medical application that can track high-risk stroke and stemi patients who need time-sensitive Emergency Medical Services (EMS) [25]. This application can be operated on both Android and iOS systems and a Global Positioning System (GPS) Tracker is used to indicate the transmitter’s real-time location. The researchers studied the use of a GPS Tracker through GPS satellites and transmissions over the 3G mobile phone network with a focus on programming to integrate them with the system for semi-automatic usage. The programme was tested in urban areas where there was Internet access to enable the system to accurately locate patients. This study provided further assurance that emergency medical services could be optimised by the application of information technology for the benefit of patient safety.

Ruangtananurak developed an emergency alert system including maps for the positioning of emergency medical services [26]. Victims or witnesses of an accident can use this application on a smart phone to send information from the scene, such as the location or other salient information, to emergency centres in the area so that the Emergency Medical Services (EMS) can quickly dispatch emergency ambulances to the right location. It also includes directions to the nearest hospital.

As can be seen from the aforementioned examples [22–26], mobile-based systems can optimise triage by enabling severe medical cases to be identified quickly for accurate and immediate intervention. However, patients should also be able to describe and determine the severity of their symptoms based on the proposed mobile application and it should contain a function to sort symptoms based on the Thai dispatch standard.

### Thai triage standard

Triage is a process that involves determining and prioritising patients’ treatment based on the severity of their condition to facilitates the efficient use of limited resources. It may also be used when patients arrive at the emergency department or in telephone medical advice systems [27] and, according to the National Institute for Emergency Medicine [28–30], it can increase the accuracy of the pre-hospital process by reducing the subjectivity of an initial diagnosis.

The criteria-based initial dispatch code used as the standard in Thailand is based on 25 main categories of patients’ signs and symptoms (National Institute for Emergency Medicine [28, 31]. This CBD protocol enables the status of patients who request pre-hospital resources to be rapidly identified as unstable or “sick” [32] by questioning them in an interview and assigning them one of five colour codes. The IDC is shown in Table 1 with details of the triage criteria and corresponding colours, while Table 2 contains the 25 main categories of symptoms. According to the CBD protocol, the results of the patient’s interview will be presented as an IDC; for example, patients will be triaged by considering each criterion of their symptoms and the result will show the main symptom code, a colour code and a triage criterion. A result of “12 red 1” represents a critical emergency patient with cardiac arrest so that the emergency staff can request emergency medical resources and operate based on the corresponding essential response.

**Table 1:** The explanation of Initial Dispatch Codes (IDC)

Colors	Triage Criteria	Essential Response
Red	Critical emergency patients	Responsible to pateint with Basic Life Support Unit: (BLS) within 4 mins after accident then responsible to pateint with Advance Life Support unit (ALS) within 8 mins after accident.
Yellow	Urgent emergency patients	Responsible to pateint with BLS within 8 mins after accident then responsible to pateint with First Response Unit (FR) within 15 mins after accident
Green	Not urgent emergency patients	Responsible to pateint with BLS within 8 mins after accident.
White	General patients	Responsible to pateint via telephone referral program and consider to BLS
Black	Not patients	No responsible to pateint

**Table 2:** The 25 main of symptom categories

Code	Symptom	Code	Symptom
1	Abdominal/Back/Groin Pain	14	O.D./Poisoning
2	Anaphylaxis/Allergic Reaction	15	Pregnancy/Childbirth/Gyn.
3	Infectious Disease	16	Seizures
4	Bleeding (Non-traumatic)	17	Sick (Unknown)/Other
5	Breathing Difficulty	18	Stroke (CVA)
6	Cardiac Arrest	19	Unconscious/Unresponsive/Syncope
7	Chest Pain/Discomfort/Heart Problems	20	Pediatric Emergencies
8	Choking	21	Assault/Trauma
9	Diabetic	22	Burns - Thermal/Electrical/Chemical
10	Environmental/Toxic Exposure	23	Drowning/Near Drowning/Diving or Water-related Injury
11	--Not define symptom--	24	Falls/Accidents/Pain
12	Head/Neck	25	Motor Vehicle Accident (MVA)
13	Mental/Emotional/Psychological	–	–

## Method

### Software development process

A medical mobile application based on CBD was developed for patient triage in Thailand to resolve the aforementioned problems associated with the pre-hospital process. The adapted waterfall methodology software was applied as a 7-step guideline for the development process [33, 34].

#### Step 1 software requirement

This step involved collecting information from emergency doctors, emergency staff and nurses in order to identify the functions necessary to triage patients.

#### Step 2 analysis

The development process involved considering the usability of the proposed application in terms of functionality, convenience, triage accuracy and accessibility. Functional requirements were identified based on the software requirement in step 1. The proposed benefits of using the application are shown in Table 3.

**Table 3:** Functionalities of the Triage mobile application

Function	Propose	How to use	Benefits	Graphical design
1.Triage	To identify IDC	Interview patient or consider patient habit by following the questions of CBD in application question by question. The result will show IDC to requesting a pre-hospital resources.	IDC can scope an appropriate requesting to pre-hospital resources. The IDC together with part of suggestion where patient waiting in pre-hospital should be transferred.	Point of design: 1.quickly accessible to triage 2.triage accuracy presentation 3.quickly accessible to a suggestion 4.direct manipulation for easy remembering 5.menu selection replace keyboard using
2.Finding responsibility of emergency care unit	To show nearby emergency care unit and a phone number	This function show emergency care unit based on Google map Application programming interface (API) with shortest transferring time consideration.	Patient or staff will have an information of emergency care unit which was ordered by transferring time from current location.	Point of design: 1.quickly accessible 2.accessible timing 3.menu selection replace keyboard using 4.reduce short term memory load
3.Patient triage log file	To check the triage information	Patient or staff can find the previous information of IDC code to identify the requesting of pre-hospital process	Log file can provide triage information to inform the pre-hospital process and confirm triage information.	Point of design: 1.practical used 2.design dialog yield closure 3.simplicity 4.adequate presentation of information
4. Exporting IDC information	To send the information of IDC to associate emergency person	Patient or staff can send doctor or provincial emergency care unit a result of IDC which contains the detail of patients’ habits from the questions of CBD.	Provincial emergency care unit can use that information for preparing emergency resources when patient is transferring.	Point of design: 1.practical use 2.convenience 3.simplicity 4.adequate presentation of information

#### Step 3 design

**Fig. 1:**
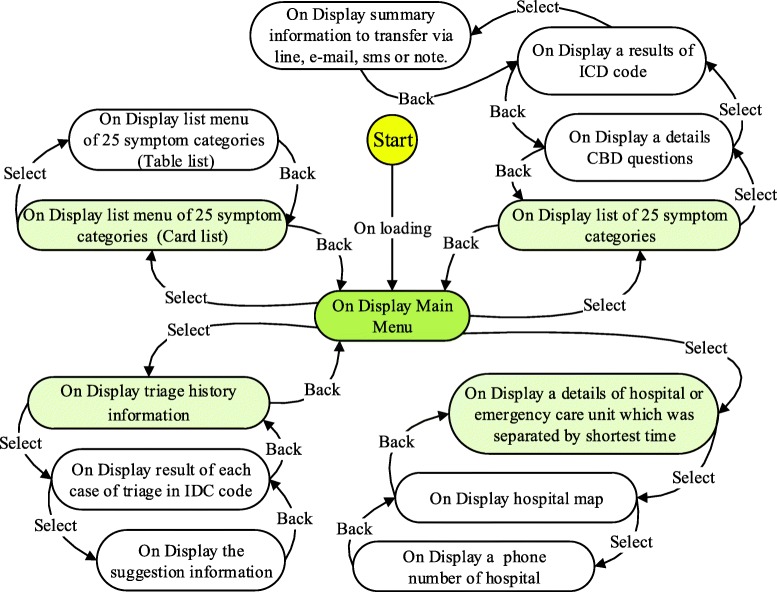
State diagram shows the functional design of application which is corresponding to functional and graphical design based on the user requirement in Table 3

This step involved using the analytical results to design the functions of the application and the graphic user interface. The functions were designed by considering the practical use of the application. State diagrams were used to describe the system’s behaviour. The functional design shown in Fig. 1 corresponds to the functional and graphical design in Table 3. In this step, the application was designed based on the requirements of a user interface on a mobile application, which included navigation components, input controls, screen proportion design, menu list navigation and a deep layout of the screen using the human-centred theory, Eight Golden Rules of interface design and Nielsen’s Ten Heuristics [35, 36].

#### Step 4 development

This step involved applying the Ionic Framework, which is a non-native language for mobile application development. This framework is suitable for both iOS and Android operating systems. Firebase was employed for the user database, which included triage results and user information, as well as the other information used in the application.

#### Step 5 testing

The advanced functions of a smartphone device that indicate its usability are its capacity to adapt (especially in medication mobile applications), users’ interaction, perception of places and objects and the interaction between the system and users, especially in cases of emergency cases. Misplacing any of these elements would result in miscommunication, which would require more operating time. Hence, in this step, the application had to be tested by triage staff to determine its performance in terms of time. Moreover, the IDC accuracy and question flow of triage had to be tested and compared to the flow of the CBC protocol by emergency voluntary staff. Therefore, the three points on which the application was tested are illustrated in Table 4.

**Table 4:** Points of consideration for the application testing

Testing Criteria	Proposes	Methods
1. CBD questions flow assessment	- Correct or incorrect IDC in each symptom	- 25 symptoms were tested and compared with step by step of CBD question -Testing by comparing with CBD handbook case by case which checked by medical emergency doctor
2. Triage time evaluation	- Time of testing for using application, open handbook and emergency medical staff.	-Testing by comparing with simulated scenario and comparison time for using application, open handbook and emergency medical staff - Using independent sample t-test analysis to compare triage time consuming between application and staff experiences
3. Application usability test	- Consider as user friendly and practical uses in real operation	- Evaluating by 10 emergency staffs who were selected as a sample group. Application testing and questionnaire were used. - Nielsen’s ten usability heuristics mixed with the human-centered theory, and Eight Golden Rules of interface design were used for testing for application performance in term of usability and user friendliness.

#### Step 6 operations

This step was taken after the verification of the ability of the application to produce an accurate CBD based on an accurate IDC by medical emergency doctors. Ethical approval for the application was requested before releasing the data to rescue teams, rescuers, community hospitals and emergency medical volunteers via Google Play and the App Store.

## Results

### Development of Mobile application

The application was developed using the Ionic Framework and evaluated based on compatibility, errors, missing information and quality. The final version that was released consisted of four main functions, including triage, finding the responsible emergency care unit, patient triage log file, and export IDC information.

The functions of the application were tested using iPhone 6 s and Huawei Android phone (P20Pro). At the time of the study, smartphone specifications were defined for medium- and large-screen display devices, due to visibility concerns [37].

**Fig. 2:**
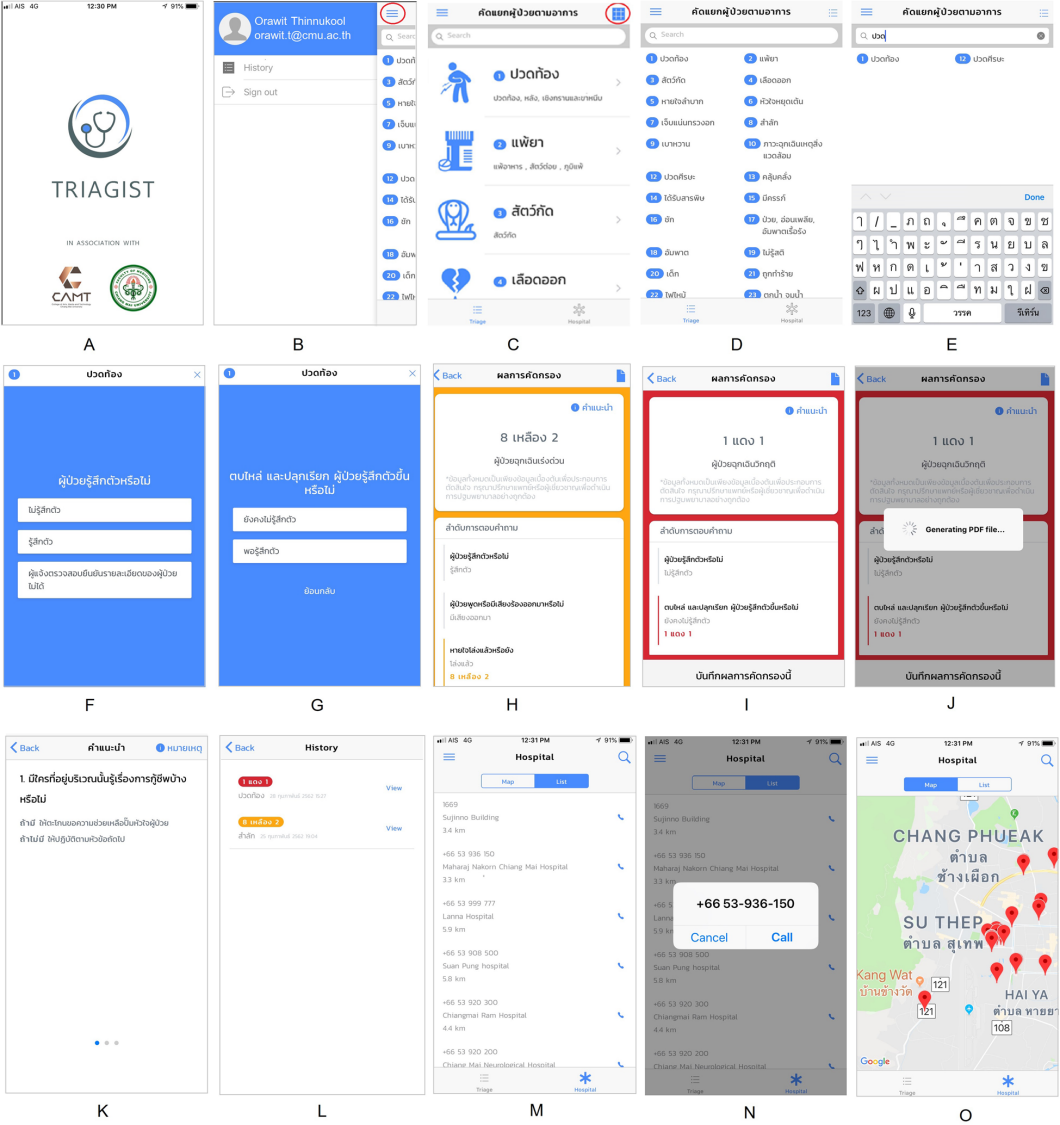
Graphic user interface of Triagist mobile application A: Main page of Application B: The main menu which provide menu list adjustment of conveniences in real operation. C: Menu in list view which present icon in each patient condition D. Menu in table view which presents icons for patient conditions E. User can search for triage types by typing a patient condition. F. Screen shot showing a question to ask patient condition and a list of answers. G: Screen shot showing the next question to ask patient condition and a list of answers. If a user is unsure about patient conditions, the user can step back to the previous questions to reconsider them. H: The triage result screen showing an IDC code (Yellow) to present the level of emergency index and the answer summary of the CBD questions. I: Screen shot showing a triage result with the suggestion button, where the user can get more information for first aid treatment or any further suggestions for the pre-hospital process including the button to record the triage result. J: Screen shot of building a pdf. File of IDC result to send to a medical unit K: A suggestion screen shot and the suggestion details L: log file of triage for history information M: Screen shot showing a list of nearby hospitals or any medical institutions (ascending sorted by the distance from the current location) N. Phone numbers are linked and enabled for click to call to initiate immediate phone calls from the application. O. Screen shot showing a map with nearby hospitals or any medical institutions

The application procedure starts when the user opens the application, and screen A in Fig. 2 appears, showing the title and welcome graphics. The main menu screen is then displayed. The buttons on screen B support the application login and triage history. Screen L contains icons for the 25 symptom symbols for triage which are demonstrated in a table view (screen D) and list view (screen C), respectively.

As shown in Fig. 2, on screens E-G, users can search by typing the symptom name or keyword(s) into the textbox and select a symptom from the list of search results. The steps for the CBD questions are shown on screen F. Users have to select their answer to go to the next question, and then continue until the IDC is displayed on screen H or I. Screen I shows the triage screening result, which identifies the IDC code and emergency status. The screen colour will be the same as the IDC code pattern. Suppose the IDC pattern is displayed in the form of “X colour Y”, the first X refers to the group number of the symptoms corresponding to the information input in Table 2 and the last Y refers to the level of urgency (1 being the highest and 9 being the lowest). On screen K, users are advised of some actions they can take while waiting for the emergency services. Moreover, they can record the IDC code via the application to summarise their condition, as well as the level of urgency. This will enable the emergency doctors/staff to gather information for the pre-hospital process. Additionally, on screen J, the application supports the export of the details of patients/emergency cases in a .pdf format to other applications, such as Line and Short Message Service (SMS). On screen M, users are able to find their nearest emergency care unit and hospital, as well as the contact information (phone numbers as shown on screen N). This data is sorted by the shortest distance and shortest time to the destination. Furthermore, a map with directions to hospital locations is provided on screen O with an overview of the information.

### CBD question flow assessment

The CBD questions used in the application were tested by evaluating the accuracy of the IDC results. All the 25 symptoms were tested in each question by following the flow in the CBD handbook. Emergency medical doctors tested the application by simulating a scenario and the results were compared with the handbook. After verifying and approving the flow of the CBD questions, the testing proceeded to the next step.

### Triage time evaluation

The application was used to triage patients in order to test its reliability and efficiency compared to various triage methodologies. 2 of the 25 symptoms, trauma and non-trauma, were tested in a simulated scenario and role-playing was used to communicate the patients’ condition to the medical staff. The accuracy of the resulting IDC was evaluated and it was confirmed to be suitable for requesting resources. This involved comparing the IDC and the triage time spent by the emergency staff in obtaining it using the above-mentioned process based on three different methods: 1) using the application, 2) following the CBD protocol from the handbook, and 3) relying on emergency medical staff’s experience. Four emergency medical staff participated in the pilot test.

The results of testing each method in each scenario are shown in Table 5. It is clear that using the application for triage was as fast as the time taken by experienced emergency staff and as accurate as following the CBD protocol from the handbook. Some IDC misrepresentations were found when performing triage by relying on the staff’s experience.

**Table 5:** Scenario test comparison for triage time testing and triage result

Patient habits	Application	Open Handbook	Experienced staff
**Scenario 1**: **Environmental/Toxic Exposure** 1.conscious or breathing 2.unable to speak normally (work of breathing)	5 s IDC: 14 Red 2 Critical Patient	13 s IDC: 14 Red 2 Critical Patient	4 s IDC: 14 Red 1 Critical Patient
**Scenario 2**: **Environmental/Toxic Exposure** 1.conscious or breathing 2.no asthma 3.breathe fast 4. age < 20	8 s IDC: 14 Red 2 Critical Patient	14 s IDC: 14 Red 2 Critical Patient	6 s IDC: 14 Red 2 Critical Patient
**Scenario 3**: **Anaphylaxis/Allergic Reaction** 1.conscious 2.breathing 3.speak normally 4.fainting 5.drug allergic	20 s IDC: 2 Yellow 4 Urgent Patient	28 s IDC: 2 Yellow 4 Urgent Patient	15 s IDC: 2 White 2 General Patient
**Scenario 4**: **Anaphylaxis/Allergic Reaction** 1.unconscious 2.responding to other 3.breathe fast 3.speak normally 4.asthma 5.age < 20	15 s IDC: 2 Red 2 Critical Patient	18 s IDC: 2 Red 2 Critical Patient	10 s IDC: 2 Yellow 8 Urgent Patient

Another test was undertaken to confirm the triage time by comparing the results based on two methods, the first of which was using the application and the second was relying on the emergency staff’s experience. Please note that the hand book was not used to evaluate the triage condition. It was only used to test the reliability of the application, since it is not a practical process for real-world operation.

The test conditions were established based on the acquisition of sample data from the 12 emergency medical staff, each of whom had no more than 5 years’ work experience. The medical staff were split into two groups of six. Triage was performed by both of these groups using the application based on their experience. The test cases included 13 out of 20 scenarios, which covered the 25 main symptom categories (See Table 2). The average triage operational time was calculated.

**Fig. 3:**
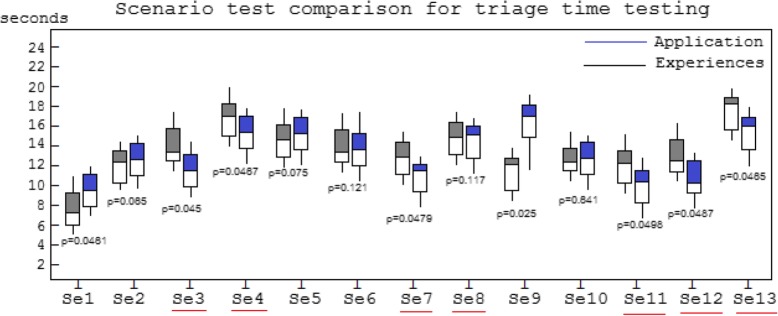
Scenario test comparison for triage time testing and triage result. The red underlined scenario represented the trauma condition (emergency patient)

The results of testing the triage time shown in Fig. 3 indicate that the use of the application was more efficient to triage non-trauma cases than relying on experienced staff; on the other hand, experienced staff were more efficient than the application in triaging trauma cases.

### Application usability test

It was important to test the usability of the application to ensure its quality and reliability. The focus had been on developing a high-quality medical system based on reliable software in order to address the practical concerns that were the pain points of the previous system. Hence, the criteria of Jakob Nielsen’s usability test technique were applied in certain scenarios. The key points tested were the occurrence of difficulties, the doubt of visual representation, doubt of usage, missing information and confusion when using the application. Each function of the application was tested by 12 emergency medical staff, including doctors and nurses. The results of the findings for each criterion (based on user motivation), as well as the application’s functionality, are shown in Table 6.

**Table 6:** Cross matrix usability test result

	User Motivation							
		Usability difficulties	Bug founding	Doubt visual representation	Doubt on usage	Missing information	Misunderstand	Total
**Function Test**	1. Triage	1	0	3	2	0	6	12^2^
	2. Finding responsibility of emergency care unit	2	0	2	0	4	1	8^3^
	3. Patient triage log file	0	0	1	3	0	3	7
	4. Exporting IDC information	3	0	2	3	0	4	13^1^
	**Total**	6	0	7^3^	8^2^	4	13^1^	22

The triage function was misunderstood in 12 instances (ranked 2nd in operational errors). Information was found to be missing when users wanted to repeat the CBD questions, which led to the duplication of the IDC of patients’ conditions in the log file. Doubt of the visual representation was found when users did not read the information shown on the left-hand side of the previous answer and it was sometimes difficult to find the answers to previous CBD questions. The second function, finding an emergency care unit, was found to be missing 4 times and this was ranked the 3rd overall operational error. This may have been due to the use of third party navigation applications because the list of available emergency care providers’ locations is displayed using navigation applications such as Google Maps. It is also possible that other errors arose from navigation applications. When testing the next function, the patient triage log file, doubt on its usage and misunderstanding were found in 3 cases. The logging file operated as a historical record of triage information to confirm the patient’s condition. The last function tested was the data export function to export IDC information to other applications, such as SMS, Line, WeChat, e-mail, etc., and its use was found to be problematic in 3 cases. Users encountered problems in sending information when the device was not connected to the internet. The function was found to be confusing in 4 cases because the users assumed that it was meant to be used to produce a medical report.

Confusion was found to be in the 1st ranking of the evaluation of users’ motivation based on a total of 13 cases. Doubt of usage, which occurred during the log file exportation of IDC information was ranked 2nd, while doubt of visual representation of some graphics and figures in the application during the triage function, finding the emergency care unit function, and the exporting of IDC information function were ranked 3rd, 4th and 5th respectively.

## Discussion

This research was conducted to respond to the question: What should a mobile medical application include to help primary care physicians or patients to screen symptoms in both normal and emergency conditions? This involved designing and developing an application that would enable patients in Thailand to determine their own symptoms and assess the need for emergency treatment.

The results were based on an application analysis and literature review from previous studies of several medical application systems [2], the emergency alert system, map positioning for emergency medical services, patient monitoring and tracking system for high-risk patients who require services from EMS [3–5, 16, 20, 25, 26] and dental application triage development [24], all of which are systems that can be used to support medical emergency operations. Hence, the proposed application was designed for the Thai social context and it may be used in the same way as other similar applications to provide patients with an alternative choice for emergency aid.

The application for patients’ triage was developed by following the CBD to request an IDC for the pre-hospital process [30–32] and it can serve as a tool for primary emergency medical practitioners and general users. The adapted waterfall methodology was used together with the Ionic framework to produce a system that can be used on both Android and iOS operating systems [33, 34]. The human-centred theory, Eight Golden Rules of user interface design and Nielsen’s Ten were employed to design an appropriate practical user interface for real emergency situations [35, 36].

The 25 main symptom categories covered by the CBD were used to design and develop the application [30] and 12 emergency medical staff, including doctors and nurses, tested the following aspects of the system: triage protocol correction, triage reliability, usability and users’ satisfaction.

Four functions were developed as a result of the proposed application: (1) the triage function to identify the IDC, (2) the finding of emergency care unit function, (3) the patient triage log file function providing triage information to support the pre-hospital process and confirm triage information, and (4) the exporting IDC information function for sending the obtained IDC to provincial emergency care units where it can be used to prepare emergency resources while the patient is being transferred.

The first application test was undertaken to check the reliability of the triage and the time spent performing triage under different conditions. This involved establishing a scenario to compare triage times. The results showed that using the application took less time than relying on experienced staff in the case of non-trauma patients, while using experienced staff to identify IDC in case of trauma patients took less time than using the application. The accuracy of identifying IDC using the handbook and the application was found to be similar.

The second test involved sampling 13 scenarios from a total of 20. In the case of trauma patients, the majority of experienced staff required less time to triage than the application, but the triage may have resulted in misrepresenting IDC. The application was shown to be useful in the majority of the scenarios in terms of operational time and reliability, but it is more likely to be suitable for use with non-trauma patients. However, Savamongkornkul found in his study that the performance of mobile applications in triage could be enhanced when they were used by experienced staff [13].

The second test was conducted as described in section 3.4, the application usability test. The testing criteria applied were the human-centred theory, Eight Golden Rules and Nielsen’s Ten Heuristics [35, 36]. The results showed that the application still had the weakness of some confusion in its use. The triage function, in which users needed to answer a number of CBD questions, was ranked the highest weak point, since some users misunderstood the number of answers required. In fact, some questions only required 1 or 2 answers.

It was also found that missing information from the finding emergency care unit function was caused by the Google API providing incorrect locations that are not emergency care units. Hence, the API usage needed to be amended in order to find the correct locations.

The testing of the application highlighted some remaining weak points based on users’ perception, but it was proved to be useful overall for patients to self-assess their symptoms correctly, including emergency situations, which will increase the effectiveness of treatment. Therefore, this project can improve the development of primary care based on designing an application that can provide medical professionals with more knowledge at the pre-hospital stage. The utility of the research can improve the performance of primary medical emergency staff based on the accuracy of the triage standard. The overcrowding of hospital centres in each Northern Province can be reduced when patients apply the triage mobile application for a primary diagnosis. As mentioned earlier, the results concomitantly support those of Savamongkornkul [13]. Also, if patients use the application as a tool to triage themselves when they experience a suspicious symptom, it can help the ED to reduce the service time and increase the quality of medical service and Kazi [11] observes that service time is related to patient outcome. Hence, this application is very suitable for patients who request CBD codes without comprehensive medical knowledge and skills.

## Conclusion

A new system was proposed and developed to respond to the problems with the system used by the National Institute of Emergency Medicine of Thailand. The Thai mobile medical application for patient triage based on the CBD was the first mobile application for triage in Thailand to support the pre-hospital process, especially for patient triage using IDC to request emergency resources. The application was designed and developed based on usability and reliability tests, which is considered to be appropriate for the Thai social context. These tests confirmed that the application is useful in terms of operational time and reliability, although it is more likely to be suitable for non-trauma rather than trauma patients. Hence, the application can be used as a practical tool as well as an educational tool for new emergency staff who lack sufficient skills in the medical emergency field.

In summary, the Thailand mobile medical application for patient triage is smart technology, which can benefit users as a tool for rapid triage screening, educate patients and new emergency staff, and prevent the overcrowding of emergency care units in Thai hospitals.

## Data Availability

The datasets used and analyzed during the current study are available from the corresponding author on reasonable request. The data are not publicly available due to privacy and/or ethical restrictions. The statistical analysis was computed by R program version 3.1.2.

## Abbreviations

ED: Emergency Department
CBD: Criteria-based Dispatch
EMD: Emergency Medical Dispatch
IDC: Initial Dispatch Code
OPD: Outpatient Department
ESI: Emergency Severity Index
EMS: Emergency Medical Services
NIEM: National Institute for Emergency Medicine of Thailand
BLS: Basic Life Support Unit
ALS: Advance Life Support Unit
FR: First Response Unit
GPS: Global Positioning System
API: Application Programming Interface
SMS: Short Message Service
